# MicroRNA Expression Changes and Integrated Pathways Associated With Poor Outcome in Canine Osteosarcoma

**DOI:** 10.3389/fvets.2021.637622

**Published:** 2021-04-15

**Authors:** Deanna D. Dailey, Ann M. Hess, Gerrit J. Bouma, Dawn L. Duval

**Affiliations:** ^1^Department of Clinical Sciences, College of Veterinary Medicine and Biomedical Sciences, Colorado State University, Fort Collins, CO, United States; ^2^Flint Animal Cancer Center, Colorado State University, Fort Collins, CO, United States; ^3^Cell and Molecular Biology Graduate Program, Colorado State University, Fort Collins, CO, United States; ^4^Department of Statistics, College of Veterinary Medicine and Biomedical Sciences, Colorado State University, Fort Collins, CO, United States; ^5^Department of Biomedical Sciences, College of Veterinary Medicine and Biomedical Sciences, Colorado State University, Fort Collins, CO, United States; ^6^Tumor-Host Interactions Research Program, University of Colorado Cancer Center, Anschutz Medical Campus, Aurora, CO, United States

**Keywords:** osteosarcoma, bone cancer, prognosis, miRNA, microRNA, predictive signature, canine (dog)

## Abstract

MicroRNAs (miRNA) are small non-coding RNA molecules involved in post-transcriptional gene regulation. Deregulation of miRNA expression occurs in cancer, and miRNA expression profiles have been associated with diagnosis and prognosis in many cancers. Osteosarcoma (OS), an aggressive primary tumor of bone, affects ~10,000 dogs each year. Though survival has improved with the addition of chemotherapy, up to 80% of canine patients will succumb to metastatic disease. Reliable prognostic markers are lacking for this disease. miRNAs are attractive targets of biomarker discovery efforts due to their increased stability in easily obtained body fluids as well as within fixed tissue. Previous studies in our laboratory demonstrated that dysregulation of genes in aggressive canine OS tumors that participate in miRNA regulatory networks is reportedly disrupted in OS or other cancers. We utilized RT-qPCR in a 384-well-plate system to measure the relative expression of 190 miRNAs in 14 canine tumors from two cohorts of dogs with good or poor outcome (disease-free interval >300 or <100 days, respectively). Differential expression analysis in this subset guided the selection of candidate miRNAs in tumors and serum samples from larger groups of dogs. We ultimately identified a tumor-based three-miR Cox proportional hazards regression model and a serum-based two-miR model, each being able to distinguish patients with good and poor prognosis via Kaplan–Meier analysis with log rank test. Additionally, we integrated miRNA and gene expression data to identify potentially important miRNA–mRNA interactions that are disrupted in canine OS. Integrated analyses of miRNAs in the three-miR predictive model and disrupted genes from previous expression studies suggest the contribution of the primary tumor microenvironment to the metastatic phenotype of aggressive tumors.

## Introduction

Despite increased survival in osteosarcoma (OS) patients resulting from the addition of chemotherapy to standard treatment protocols, only about one-fourth of canine OS patients will survive longer than a year (1). New treatment strategies are needed to manage this disease and will likely include integration of targeted therapies with standard chemotherapeutics in an individualized medicine setting. To facilitate this effort, biomarkers of disease progression and response to treatment are needed to optimize the stratification of patients into groups most likely to benefit from various treatments and identify targets for development of novel therapeutics.

Previous gene expression studies in our laboratory identified the activation of the Notch signaling pathway in OS but suggested that Notch-independent changes in HES1expression resulted in low HES1 expression in the most aggressive tumors. We also identified upregulation of insulin-like growth factor 2 mRNA binding protein 1 (IGF2BP1), an oncofetal protein and known target of the let-7 tumor suppressor family of miRNAs that has been implicated in various cancers (2–4). We hypothesized that the disconnect between the HES1 and Notch pathway activation, as well as the escape of IGF2BP1 from inhibitory mechanisms present in normal adult cells, likely involved the disruption of post-transcriptional regulation by miRNAs.

miRNAs are small non-coding RNAs involved in the regulation of gene expression, providing fine-tuning of multiple cellular processes involved in the development and maintenance of homeostasis. In general, miRNAs suppress the expression of their target genes, and it is estimated that half of mammalian genes are subject to miRNA regulation via 3′ UTR binding sites (5, 6). Since a 2002 report from the Croce laboratory, the involvement of miRNA dysregulation in cancer has been well-established (7). Molecular genomic techniques such as cDNA microarrays and next-generation sequencing have been adapted to facilitate miRNA expression biomarker and novel target discovery efforts (8, 9).

A growing body of literature exploring the significance of miRNA expression changes in OS exists. Several comprehensive reviews have been written to summarize the involvement of miRNAs in OS (10–18). Major findings in OS miRNA studies include suggested or experimentally demonstrated oncogenic- or metastasis-promoting roles for miR-17-92 cluster (19–21), miR-181 family (22–24), miR-27a (23), and miR-21 (25, 26) as well as tumor-suppressive roles for miR-15/16 family members (23) and miR-34 (20, 27, 28). The roles of other miRNAs are less clear, such as the miR-29 family with reports of both elevated and decreased expression in osteosarcoma cell lines and tumors compared to “normal” controls for each sample type (20, 21, 23, 24). Significant bodies of work have explored the association of miRNAs in OS with prognosis. Loss of miRNAs located in the 14q32 locus has been associated with poor patient outcome in both human and canine OS, with the findings in human OS confirmed by multiple groups (29–31). In addition to confirming the oncogene- and tumor-suppressive roles of mir-27a and mir-16, respectively, both *in vitro* and *in vivo*, Jones et al. (23) identified tumor-based signatures associated with “osteosarcomagenesis,” metastasis, and response to chemotherapy. Several reports have included functional experiments confirming interactions between miRNAs of interest and genes previously identified as dysregulated in OS, such as loss of 14q32 miRNAs and miR-135 with upregulation of c-MYC, miR-34 with RUNX2, and miR-20a of the miR-17-92 cluster and Fas (19, 28, 31, 32).

miRNAs are attractive molecules for biomarker discovery efforts due primarily to increased stability in biologic fluids and in formalin-fixed tissues compared to other RNA molecules (33–35). These features exemplify the clinical utility of miRNA, particularly in healthcare settings where stringent sample collection and storage requirements necessary for the analysis of mRNA are not always possible. A handful of studies focusing on human OS have identified associations between miRNA expression and outcome, including studies utilizing paraffin-embedded, formalin-fixed tissues, and blood fluids (23, 29, 36). Consequently, we explored the hypothesis that cancer-associated miRNAs would be measurable in tumor and serum and associated with outcome.

Our first objective was to identify candidate biomarker miRNAs differentially expressed in tumors from different outcome groups and in all tumors relative to normal bone. Candidate miRNAs were measured in a larger group of tumors and similarly sized set of serum samples to determine associations between miRNA expression changes and patient outcome. Finally, pathway and miRNA target prediction analyses were used to integrate miRNA and gene expression data to identify potential miRNA–gene regulatory networks important for OS progression.

## Materials and Methods

### Patient and Tissue/Fluid Selection

Tumors in disease-free interval (DFI) cohorts from dogs with DFI >300 or <100 days treated with limb amputation followed by doxorubicin or platinum-based chemotherapy were collected as previously described (2). Normal bone was obtained from dogs with osteosarcoma from limbs post-amputation and harvested so that “normal” bone included in the study was distant from the tumor site and separated from the tumor by a joint (e.g., a femoral tumor would have matched a distal tibia bone collected). A 1–2-cm section of normal bone was collected for each sample; marrow and medullary fat were removed at collection. Supplementary Table 1 shows patient data for these groups of tumors. Thirty-three additional tumors were selected from the Colorado State University Flint Animal Cancer Center's tissue archive with post-treatment data to document disease progression and matched serum or plasma samples available for miRNA extraction and expression analysis (Supplementary Table 2, COS33). Dogs from both cohorts were confirmed to be free of metastatic lung disease at diagnosis and surgery. Following RNA extraction and quality checks of the serum or plasma samples from the second cohort, 24 of these patients were included in circulating miRNA expression analysis.

### Total RNA Isolation, Quantification, and Quality Assessment (Tissues)

RNA was extracted from frozen samples using a freeze fracture device, followed by homogenization and separation of RNA from DNA and protein fractions using TRIzol® Reagent (Life Technologies, Grand Island, NY). The freeze fracture device and the samples were placed in liquid nitrogen to chill for 15–20 min. Approximately 1 cm^3^ of tumor tissue and up to 4 cm^3^ of normal metaphyseal bone were used for RNA extraction. Pulverized tissue was transferred into 2 ml/cm^3^ of tissue of TRIzol in 15-ml conical tubes. The tissue/TRIzol mixture was then homogenized at medium to high speed for 1 min. Homogenized samples were gently shaken, centrifuged for 1 min at 2,000 RPM, and then incubated for 5 min at room temperature. The supernatant was collected into two 1.5-ml tubes and carried forward using the TRIzol reagent manufacturer's protocol for RNA extraction.

After resuspension of the extracted RNA pellet in nuclease-free water, the mirVana™ miRNA extraction kit (Life Technologies, Grand Island, NY) was used for additional RNA purification. RNA was eluted in 50 μl nuclease-free water and treated in 20-μl batches with DNAse (2 μl 10 × DNAse buffer and 2 μl DNAse-I; DNA-free™ kit, Life Technologies) to eliminate genomic DNA contamination. RNA concentration and purity were determined using the NanoDrop 1000 spectrophotometer (NanoDrop Products, Thermo Scientific, Wilmington, DE). The quality of isolated total RNA was determined by RNA integrity number using a Bioanalyser 2100 (Agilent Technologies, Santa Clara, CA) with a RNA 6000 Nano chip. Only samples with RNA integrity number >6 were used. All samples were stored at −80°C.

### Total RNA Isolation (Serum)

Archived serum samples stored at −80°C were thawed at room temperature, transferred to RNAse/DNAse Free 2-ml microcentrifuge tubes, and centrifuged for 5 min at 4°C and 16,000 × g. Exactly 200 μl of the supernatant was moved to a fresh 2-ml tube for extraction of RNA using the miRNeasy Serum/Plasma Kit (Qiagen, Valencia, CA) following the manufacturer's directions. Synthetic ce-miR-39 mimic (1.6 × 10^8^ copies) was spiked in to each sample prior to addition of chloroform. Strict preset volumes of reagents and sample RNA were used following the manufacturer's recommendations.

### Real-Time Reverse Transcriptase Quantitative PCR

cDNA synthesis of small non-coding RNAs was performed using the miScript Reverse Transcription kit (Qiagen, Valencia, CA) following the manufacturer's instructions. Briefly, reverse transcription (RT) was performed in 20-μl reactions containing 1 μg total RNA in nuclease-free water, 5 × miScript RT Buffer (Mg, dNTPs, and oligo-dTprimers), and 1 μl miScript Reverse Transcriptase Mix [poly(A)polymerase and reverse transcriptase]. Generated cDNAs were stored at −20°C until analysis. Quantitative PCR measurements were performed in 384-well PCR plates in a 6-μl reaction containing 2 × Quantitect SYBR Green master mix (Qiagen, Valencia, CA), 10 μM miRNA-specific forward primer (MWG Biotech), 10 × Universal Reverse Primer (Qiagen, Valencia, CA), 2 ng equivalent cDNA, and nuclease-free water. miRNA-specific primers were designed based on sequences of mature miRNA from MirBase (Supplementary Table 8). Samples were run in duplicate with non-template and reverse transcriptase-free (no RT) controls.

Modifications to this protocol for measurement of serum miRNA were as follows: cDNA synthesis was carried out in 10-μl reactions containing 2 μl 5 × HiSpec Buffer, 1 μl Nucleics mix, 1 μl nuclease-free water, and 5 μl total serum RNA. The serum cDNA was diluted 1:10 in nuclease-free water, and a consistent volume (0.15 μl), rather than a consistent concentration, was included in each 6-μl RT-qPCR reaction.

### Data Analysis

For analysis of RT-qPCR data from tumor samples, both GeNorm (37) and NormFinder (38) were used to identify the best candidate reference miRNAs from 10 options, and data was normalized to the geometric mean of miR-30a, miR-27b, and miR-185. The 2^−ΔΔCt^ method was used for differential expression analysis in the initial set of 14 tumors. Statistical analysis of survival data was performed using normalized and transformed expression data from 19 miRNAs in the test set of 33 tumors and 13 miRNAs in 31 serum samples using a combination of Prism and the coxph and survfit functions from the survival package in R.

### Statistical Analysis

Associations between miRNA expression levels and DFI were evaluated using Cox proportional hazards linear regression. Multivariable Cox regression was then performed on a subset of candidate miRNAs (*p* < 0.25 from univariate analysis), utilizing both forward and backward stepwise models based on the Akaike information criterion (AIC). A risk score was calculated for each sample based on the best multivariate model, and Kaplan–Meier method was used to determine median DFI for low- and high-risk groups based on the median risk score. Comparison between groups was made with the log rank analysis, and a *p*-value of < 0.05 was considered significant.

This analysis pipeline was modified slightly for serum samples. Raw Ct values were first adjusted based on the expression of synthetic cel-miR-39 (33, 39). Then, two miRNAs, miR-16 and miR-21, were selected as reference miRNAs and normalized using a variation of mean centering, termed concordance correlation restricted, as described in Wylie et al. (40). This method was found to be well-suited for biofluid samples.

## Results

### Differentially Expressed miRNAs in Tumors From Dogs With Poor Response Compared to Those With Good Response

Expression of 188 miRNAs was measured in 14 tumors—seven tumors from dogs with DFI >300 days (good responders) and seven tumors from dogs with DFI <100 days (poor responders)—using RT-qPCR. Four miRNAs were differentially expressed in tumors from poor responders relative to those from good responders using a cutoff of *p* < 0.05 for significance. Nineteen miRNAs were selected based on *p* < 0.1, fold change >2.0, or biological interest based on human OS studies for additional expression analyses in a larger set of tumors (Supplementary Table 3, bold).

Cox proportional hazard univariable regression analysis of expression of 19 miRNAs in 33 tumors from patients with DFI ranging from 20 to 937 days identified miRNAs associated with patient outcome (Table 1). The goal of multivariate Cox proportional hazard analysis in this study was to identify the best combination of candidate miRNAs whose expression explained a significant proportion of the variability of patient outcome in this group of tumors and which would be likely to predict outcome in an independent set of canine tumors. Thus, expression values for seven miRNAs with *p* < 0.25 based on the univariate analysis were included in both forward and backward step-wise multivariate Cox proportional hazard regression analysis. A three-miRNA model was selected as the best model based on AIC, a measurement of model selection that takes into account the goodness-of-fit of the model with penalties for increased complexity (Table 2).

**Table 1:** Results of univariate Cox proportional hazard regression analysis for expression of miRNAs in canine osteosarcoma tumors (*n* = 33; disease-free interval range, 20–937 days).

**miRNA name**	***p*-value**	**HR**	**95% CI**
*mir.223.3p*	*0.001*	*2.25*	*1.38–3.68*
*mir.181b.5p*	*0.028*	*0.65*	*0.44–0.96*
*mir.130a.3p*	*0.107*	*0.70*	*0.46–1.08*
*mir.199a.5p*	*0.158*	*0.75*	*0.50–1.12*
*let.7b.5p*	*0.171*	*0.60*	*0.29–1.25*
*mir.451a*	*0.194*	*1.24*	*0.90–1.71*
*mir.7.5p*	*0.236*	*1.22*	*0.88–1.69*
mir.26a.5p	0.315	0.80	0.52–1.24
mir.30c.5p	0.369	0.85	0.61–1.20
mir.142.3p	0.423	1.19	0.78–1.83
mir.206	0.583	0.91	0.65–1.27
mir.18a.5p	0.617	1.10	0.76–1.58
mir.16.5p	0.648	0.93	0.67–1.29
mir.196b.5p	0.668	0.92	0.63–1.35
mir.9.5p	0.742	0.94	0.65–1.36
mir.135a.5p	0.788	0.96	0.69–1.32
mir.128.3p	0.796	0.96	0.70–1.32
mir.210.3p	0.964	1.01	0.70–1.46
mir.17.5p	0.981	0.10	0.73–1.35

*The italicized rows (p < 0.25) were selected for multivariate analysis*.

**Table 2:** Three-miRNA model with lowest Akaike information criterion *via* both forward and backward step-wise Cox proportional hazard regression (*R*^2^ = 0.413, concordance = 0.73).

**miRNA name**	***p*-value**	**HR**	**95% CI**
mir.223.3p	0.0003	2.676	1.57–4.57
mir.130a.3p	0.0229	0.5718	0.35–0.93
let.7b.5p	0.1451	0.6034	0.31–1.19

### Three-miRNA Signature for Patient Outcome (DFI)

**Figure 1:**
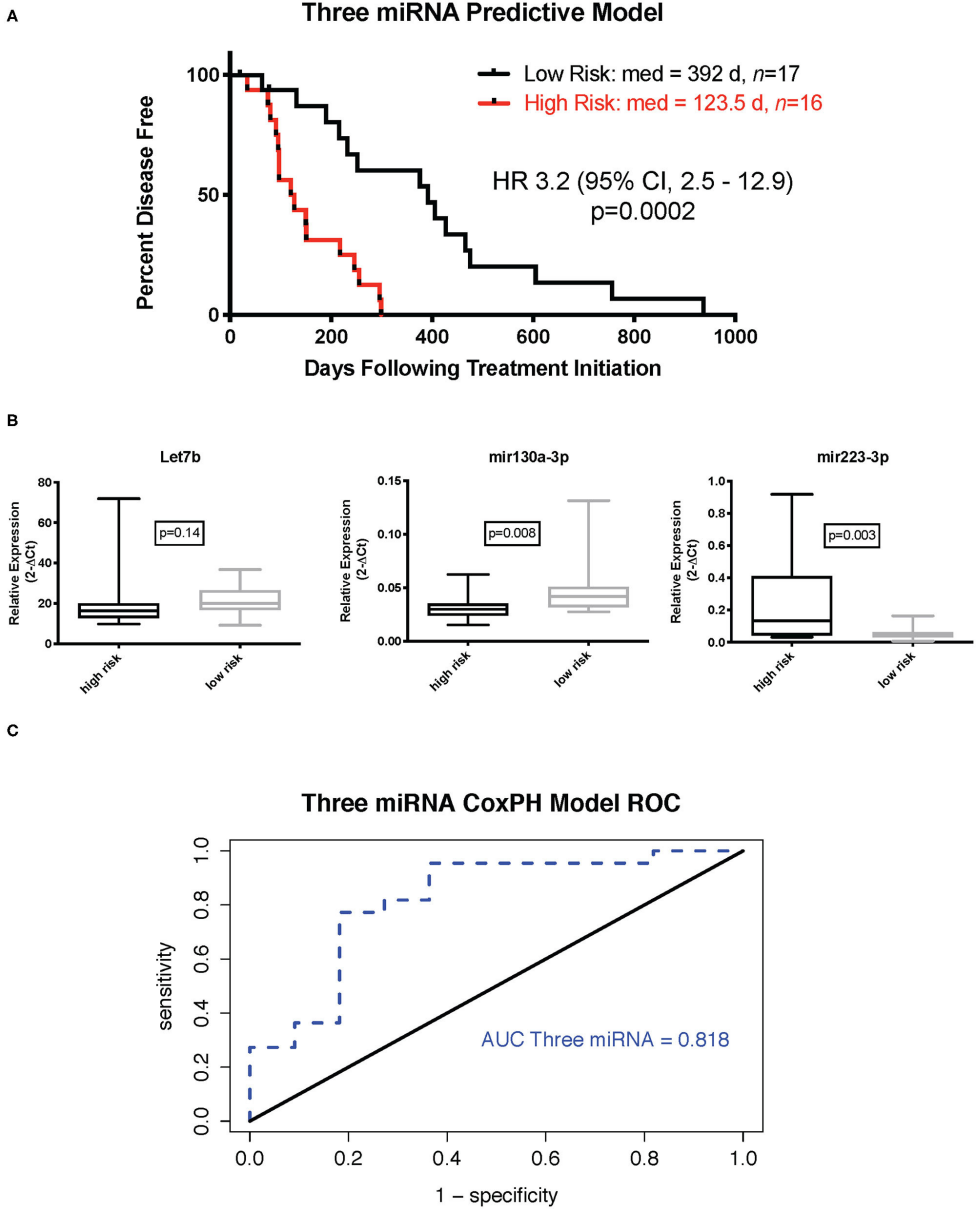
Three-miRNA tumor-based predictive model. **(A)** Kaplan–Meier survival curve with log rank test (cutoff is median risk score: 0.8897). **(B)** Relative expression (2^−ΔCt^) of individual miRNAs in low- and high-risk groups (Mann–Whitney test). **(C)** Receiver operator characteristic curve for three-miRNA Cox proportional hazard-based risk score dividing outcome groups based on mean disease-free interval.

The Cox proportional hazard multivariate model with three miRNAs—miR-223-3p, miR-130a-3p, and let-7b-5p—was used to calculate the risk scores for each sample. The median risk score was used as a cutoff to discriminate samples considered high or low risk. Kaplan–Meier survival analysis with the log rank test using the three-miRNA model-based risk score distinguished patients with high risk and low risk with respective median DFIs of 123.5 and 392 days (Figure 1A, *p* = 0.0002, hazard ratio 3.2, 95% confidence interval 2.5–12.9). Relative expression of each miRNA in the signature (Figure 1B) indicated that Let-7b was not significantly elevated in the low-risk group, while miR-103a was significantly elevated in the low-risk group (*p* = 0.008), and miR-223 was significantly reduced in the low risk group (*p* = 0.003). Additionally, if samples were separated into cohorts of good and poor responders based on mean DFI, the three-miRNA model signature had an accuracy, based on area under the curve of 0.86 (Figure 1C).

### Pathway Analysis of Dysregulated miRNAs and Genes Suggests Roles for Tumor Microenvironment and IGF2BP1 Regulatory Network in Aggressive OS

We used the mirPath tool from the Diana Tools website (41, 42) with species set to human to identify the top pathways enriched for genes that are targets of the three miRNAs in our Cox proportional hazards model. The top 20 significant pathways using the microT-CDS database and the genes union function include: FoxO signaling, ECM–receptor interaction, signaling pathways regulating pluripotency of stem cells, TGF-beta signaling, cytokine–cytokine receptor interaction, and p53 signaling (Table 3). The let-7 family, being among the earliest miRNAs discovered and more widely studied, shows 682 targets in this tool, while miR-223 and miR-130a list only 367 and 552 genes, respectively. Since loss of let-7b-5p and mir-130a-3p was associated with a shorter DFI, we explored the pathways that they regulate separately from those pathways regulated by mir-223-3p which was elevated in the tumors with higher risk of metastasis. Mir-223-3p is specifically involved in transcriptional misregulation in cancer and cytokine–cytokine receptor interaction. Taken together, this suggests a role for these miRNAs in the regulation of the extracellular environment, immune system, and developmental pathways.

**Table 3:** Top pathways (*p* < 0.05) enriched for genes targeted by let-7b-5p, miR-223-3p, and/or miR-130a-3p.

**KEGG pathway**	***p*-value**	**^#^genes**	**^#^miRNAs**
Prion diseases	4.76 × 10E-19	1	1
Mucin type O-glycan biosynthesis	3.83 × 10E-16	9	3
FoxO signaling pathway	9.91 × 10E-05	27	3
Extracellular matrix–receptor interaction	9.91 × 10E-05	12	3
Signaling pathways regulating pluripotency of stem cells	1.02 × 10E-04	29	3
TGF-beta signaling pathway	9.52 × 10E-04	19	3
Cytokine–cytokine receptor interaction	3.86 × 10E-03	30	3
Amoebiasis	0.011	16	3
p53 signaling pathway	0.046	13	3
Transcriptional misregulation in cancer	0.048	28	3

# *genes, number of genes targeted by analyzed miRNAs in the pathway*.

# *miRNAs, number of analyzed miRNAs that have targets in the pathway*.

**Figure 2:**
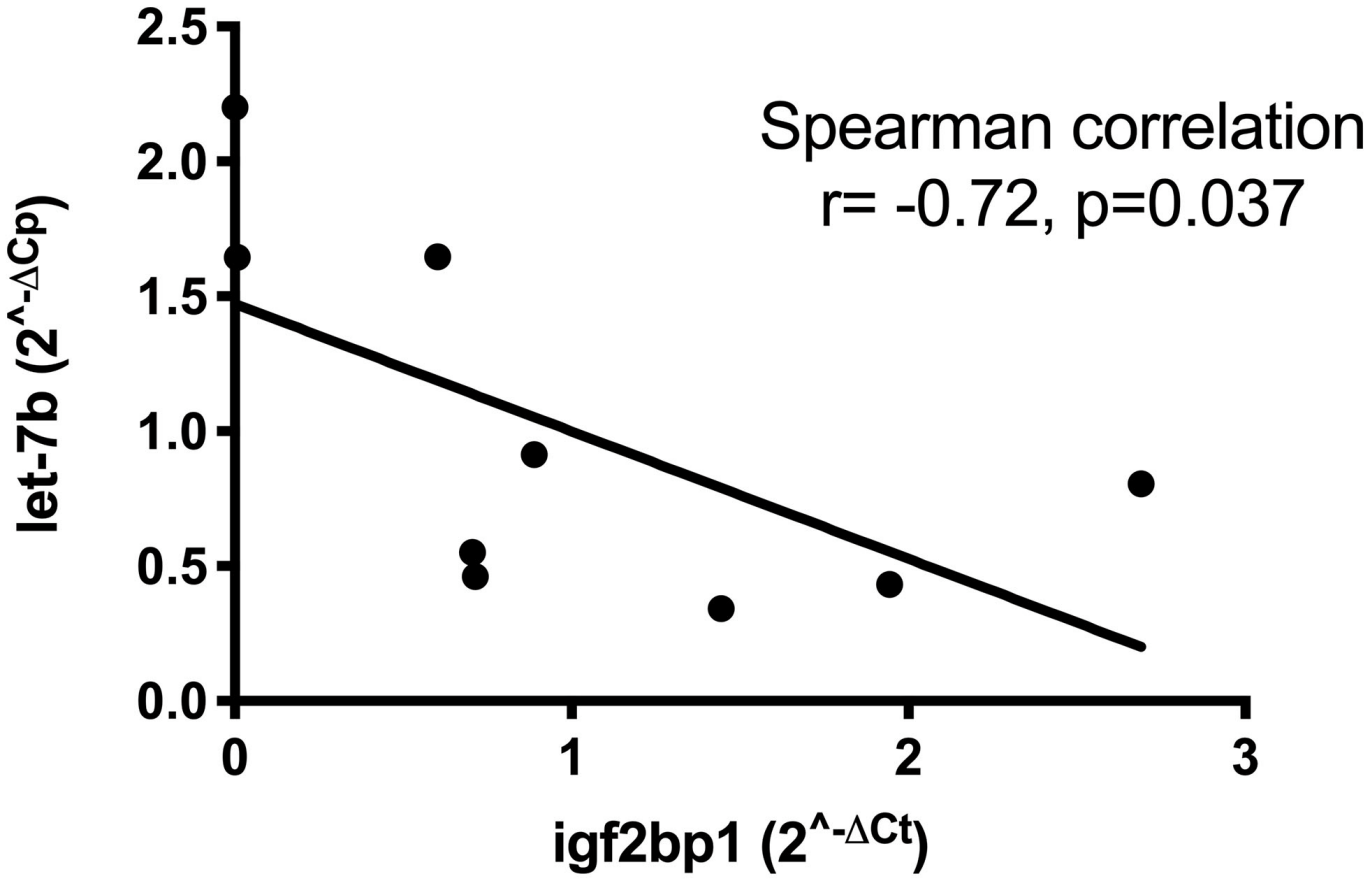
Correlation between low let-7b expression and high expression of IGF2BP1 in eight osteosarcoma tumors as determined by RT-qPCR.

We next used multiMiR, a miRNA–target interaction R package and database out of the Theodorescu lab (43), to identify either experimentally validated or predicted miRNA–mRNA interactions based on data from this study and previous studies in our laboratory (Supplementary Table 6). MultiMiR predicted potential interactions between miR-223, over-expressed in tumors from dogs with shorter DFI, and both dystonin (DST) and catenin (cadherin-associated protein), Alpha 2 (CTNNA2). Both are adhesion proteins interacting with the cytoskeleton, potentially implicating disruption of the tumor microenvironment in the aggressiveness of OS. Interactions between let-7b and six other under-expressed miRNAs and IGF2BP1 confirm that miRNA expression changes likely play a role in the high expression of this gene in tumors from dogs with poor outcome. Relative expression of let-7b and IGF2BP1 in OS tumors with a short disease-free interval via RT-qPCR confirms a statistically significant correlation, suggesting that this interaction occurs in canine OS and may contribute to outcome (Figure 2).

### Differentially Expressed miRNAs in OS Tumors Relative to Normal Bone Support Dysregulation of the Notch Pathway in OS

Expression of 188 miRNAs was also measured via RT-qPCR in seven normal bone samples. As has been our experience with gene expression, more differentially expressed miRNAs were identified with higher statistical significance and larger fold changes. Forty differentially expressed miRNAs were identified using cutoffs of *p* < 0.05 for significance and fold change >2; 21 miRNAs had a lower expression in tumors than normal bone, while 19 miRNAs were over-expressed in tumors (Supplementary Tables 4, 5).

**Figure 3:**
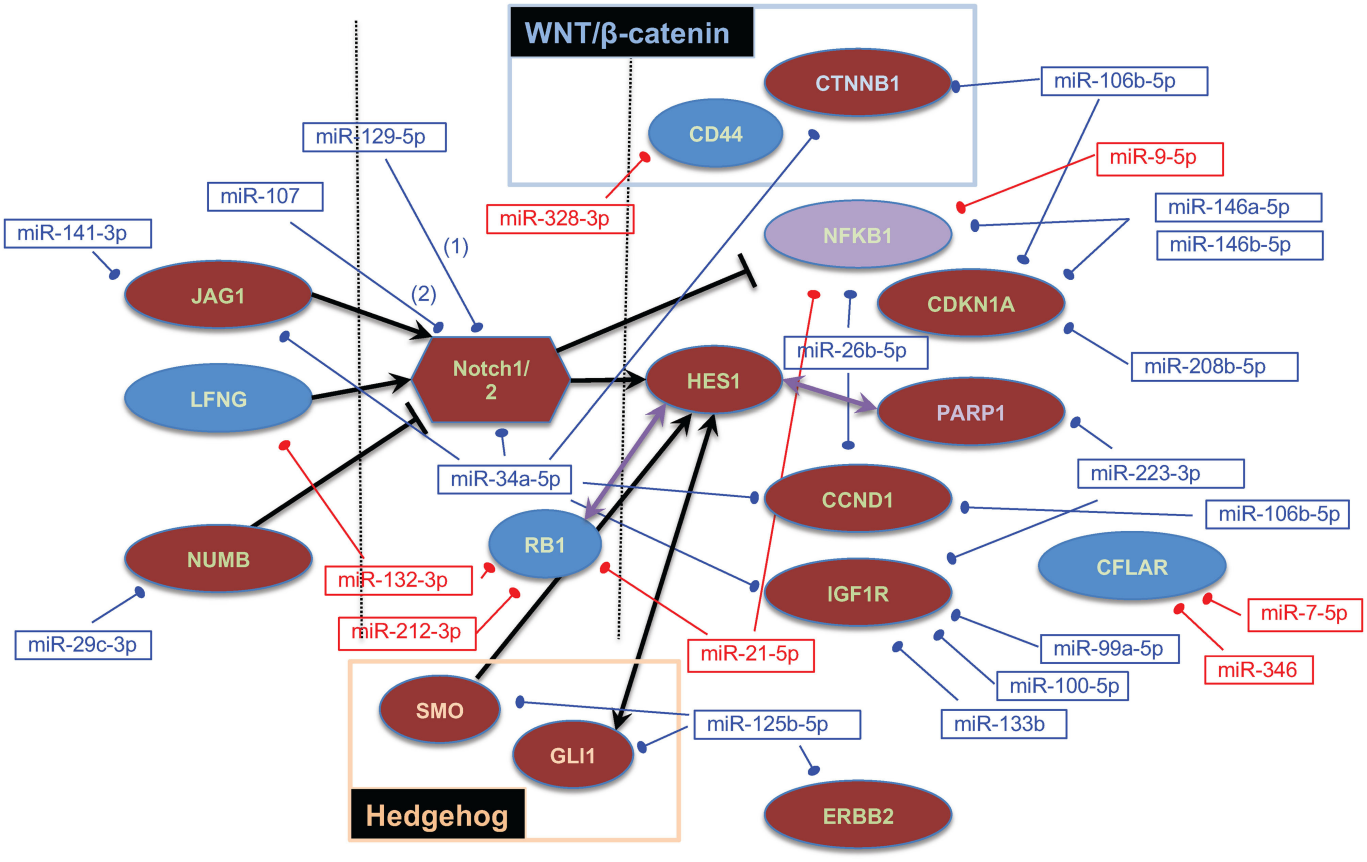
Notch/HES1-associated miRNA–mRNA interactions. Dysregulated genes are shown as ovals or polygons, dysregulated miRNAs are shown in text boxes. In both cases, red indicates expression that is higher in tumors than in normal bone, blue indicates expression that is lower in tumors, and purple indicates that one probe in the Affymetrix array showed NFKB1 as upregulated and another as downregulated. Genes on the left are ligands or inhibitors of Notch; genes on the right are downstream targets of the Notch signaling pathway and/or specifically interact with HES1.

Based on our previous work associating the Notch signaling pathway with OS and again using multiMiR, we sought validated interactions between 21 downregulated miRNAs and 30 upregulated Notch/HES1-associated genes as well as between 19 upregulated miRNAs and 14 downregulated Notch/HES1-associated genes. The pool of Notch/HES1-associated genes was a subset of the genes previously published (44). MultiMiR identified experimental, protein-based evidence for interactions between 21 of 41 miRNAs and 17 of 44 genes or roughly half of the miRNAs and genes entered into the analysis (Figure 3). This data supports the hypothesis that dysregulation of the Notch signaling pathway contributes to the pathogenesis of OS and likely involves disruption of miRNA regulation of Notch pathway-associated genes.

### Serum miRNA Changes Associated With OS Patient Outcome

**Figure 4:**
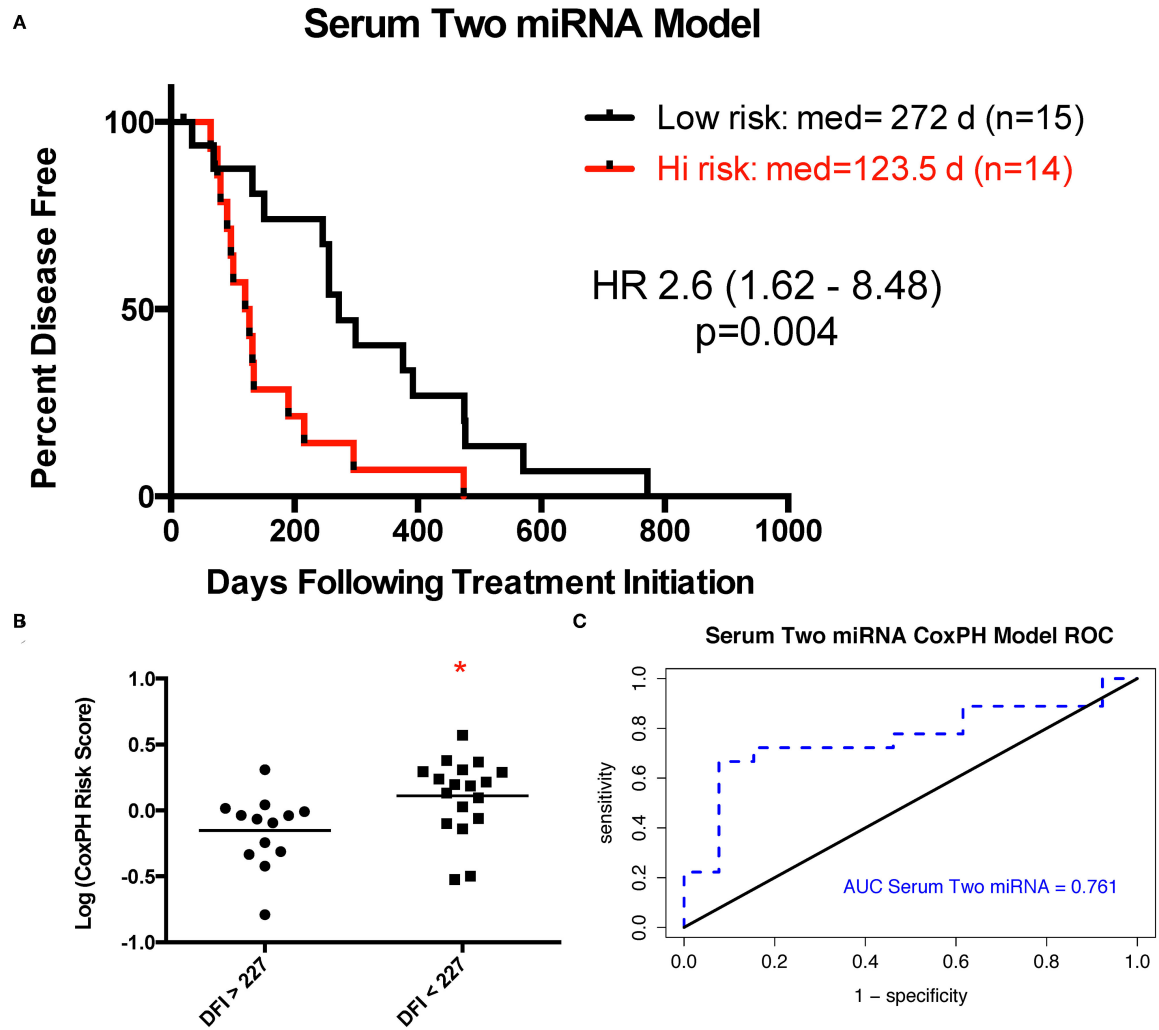
Two-miRNA serum-based predictive model. **(A)** Kaplan–Meier survival curve with log rank test (cutoff is median risk score: 1.0372). **(B)** Scatter plot of risk scores in two outcome groups based on mean disease-free interval (DFI) for all 33 samples (*p = 0.014, Mann–Whitney test). **(C)** Receiver operator characteristic curve for serum two-miRNA Cox proportional hazard-based risk score dividing outcome groups based on mean DFI.

Expression of 13 miRNAs in 31 serum samples from patients with DFI ranging from 20 to 772 days was analyzed using a similar Cox proportional hazard regression pipeline described for tumor miRNA expression data. The 13 miRNAs evaluated comprised a combination of 10 miRNAs selected from our analysis of tumor-derived miRNA expression and three miRNAs commonly highly expressed in human serum samples. Forward and backward stepwise Cox multivariable proportional hazard regression analysis identified a two-miRNA model (miR-23a-3p and miR-30c-5p) with the best fit based on AIC (Table 4, Figures 4A–C). The risk score based on this model separated the samples into groups, with mean DFI of 272 days for the low-risk group and 123.5 days for the high-risk group (*p* = 0.004, hazard ratio 2.6, 95% confidence interval 1.6–8.5).

**Table 4:** Two miRNA models after step-wise Cox proportional hazard regression (*R*^2^ = 0.278, concordance = 0.69).

**miRNA name**	***p*-value**	**HR**	**95% CI**
mir.23a.3p	0.0209	0.5652	0.35–0.92
mir.30c.5p	0.0099	0.5487	0.35–0.87

### Tumor-Based miRNA Signature Compared to Clinical Predictors

One measure of the value of a new prognostic biomarker is its usefulness compared to other predictive markers including clinical parameters (45). For OS, the most consistent clinical indicators of outcome are proximal humerus location, weight, and serum ALP (1, 46–48). We had access to an expanded set of curated, quality-checked clinical data for a subset of our tumors (*n* = 24) that were included in a large retrospective study by Selmic et al. (46). Multivariate Cox proportional hazard regression of the three miRNA expression-based risk score and other clinical parameters (*p* < 0.25 on univariate analysis) showed that, when adjusting for these indicators, the miRNA expression based risk score remains a significant predictor of outcome (Table 5). This suggests that incorporation of miRNA expression signatures would improve the estimation of prognosis for canine patients.

**Table 5:** Results of univariate/multivariate analysis of factors associated with clinical outcome, including a three-miRNA expression-based risk score (tumor-derived miRNA expression).

		**Med DFI (days)**	**HR**	***P***	**95% CI**
**Univariate analysis**					
Three-miRNA risk score	Low	392	0.18	0.00061	0.070–0.484
	High	123.5			
Weight			1.05	0.046	1.001–1.103
Age at diagnosis			0.785	0.10	0.587–1.051
Proximal humerus	Yes		3.055	0.057	0.969–9.628
	No				
**Multivariate analysis**					
Three-miRNA risk score			0.185	0.0067	0.055–0.626
Proximal humerus			5.63	0.016	1.38–23.06

## Discussion

Aberrant miRNA expression patterns have been associated with patient outcome for a variety of human tumors. Combined with their stability in fixed tissues and less invasively obtained body fluids, miRNAs make attractive candidates for biomarker discovery efforts. In this study, we identified miRNA expression signatures from both canine OS tumor and patient serum samples that associated significantly with outcome following surgical amputation of the affected limb and standard-of-care chemotherapy. Pathway and miRNA–gene interaction analyses focused on tumor-derived miRNAs associated with poor outcome, suggesting that the interaction between OS cells and the primary tumor microenvironment may be a major determinant in the ultimate metastatic capabilities of OS tumor cells. Additional miRNA–gene interaction analyses combining expression changes identified in this study with gene expression changes from earlier studies suggest that miRNA dysregulation contributes to both (1) disruption of the Notch pathway in OS compared to normal bone and (2) deregulation of the growth-promoting oncofetal protein IGF2BP1 in the most aggressive OS tumors. Finally, we demonstrated that the tumor-based three-miRNA signature remains an independent predictor of outcome when we control for possible effects of other clinical parameters such as tumor location, patient weight, and age at diagnosis.

Although previous studies have established grading systems for canine OS (49, 50), a limitation of the current study is the lack of grading for the tumors in this data set. Meta-analysis and direct comparisons have shown limited utility for these grading systems in prognosis with simplified high- or low-grade models suggested (48, 51). Variability within tumors as well as the complexity of the criteria in the proposed grading schemes may contribute to high subjectivity. Furthermore, more than 80% of tumors will fall into high-grade histologic categories, within which variable patient outcomes may be achieved. For OS, the most consistent clinical indicators of outcome are proximal humerus location, weight, and serum ALP (1, 46–48). Among these clinical parameters (scoring *p* < 0.25 on univariate analysis), only location of the tumor in the proximal humerus and the three-miRNA risk score were significant predictors of disease outcome. This suggests that incorporation of miRNA expression signatures would improve the estimation of prognosis for canine patients.

Our first goal of this study was to identify miRNAs associated with progression of OS despite standard-of-care treatment including surgical amputation and doxorubicin and/or platinum-based chemotherapy. We identified a three-miRNA expression signature that separated patients into two distinct outcome groups. Within this signature, elevated expression of miR-223 and decreased expression of let-7b and miR-130a were associated with increased risk and ultimately shorter median DFI. Of these three, miR-223 was the most significantly associated with DFI based on *p*-value in both the univariate and multivariate regression analyses. Interestingly, the expression of miR-223 is nearly 20 times lower in OS tumors compared to normal bone (Supplementary Table 5), which is consistent with two reports in human OS (21, 23). miRNA expression analyses performed in canine cancer cell lines conducted in our laboratory showed that miR-223 expression is similarly uniformly low across canine osteosarcoma cell lines. We performed pathway and miRNA–gene regulatory analyses to identify pathways potentially affected by expression changes in miR-223. These analyses suggest that the significant increase in miR-223 expression in canine OS tumors may be either originating from or influenced by interactions with the tumor microenvironment.

Pathways enriched for both miR-223 and miR-130a included hematopoietic cell development and osteoclast differentiation. Several lines of evidence support a role for miR-223 as an important regulator of the immune response inhibiting the differentiation of classically activated (M1) macrophages and promoting anti-inflammatory and pro-tumor (M2) polarization (52–54). Notch signaling, a pathway we have found to be significantly dysregulated in aggressive canine OS, is also important for pro-inflammatory M1 polarization (55). Normal differentiation and function of osteoclasts, which are derived from bone marrow monocyte precursors, are also reliant on the expression of miR-223 (56). Given that miR-223 is highly expressed by both M2 macrophages and osteoclasts, it is possible that the increased expression of miR-223 in tumors from poor responders is originating from or induced by an interaction with the increased numbers of these cells in the tumor microenvironment. For example, Yang et al. (57) demonstrated that M2-polarized macrophages can shuttle miR-223 via exosomal transport to breast cancer cells, increasing their invasive ability. In addition, miR-223 may suppress the maturation and immunogenicity of dendritic cells to promote a tolerogenic environment (58, 59).

The role of both osteoclasts and macrophages in OS remains controversial due to a variety of factors, including the potentially different behaviors of these cells depending on the level of differentiation, polarization, and response to external stimuli (60–63). Despite this uncertainty, macrophage-activating agents (promoting pro-inflammatory M1 polarization), such as muramyl tripeptide phosphatidylethanolamine, have consistently shown promise for treatment of OS (64, 65).

In further support of the influence of the tumor microenvironment on miRNA expression changes in OS, miRNA–gene interaction analysis identified potential interactions between miR-223 and adhesion proteins DST and CTNNA2. Both are involved in actin cytoskeletal remodeling, a pathway commonly associated with metastasis (66) and identified as enriched for dysregulated genes in our previous gene expression studies (2). Changes in actin cytoskeletal remodeling are commonly triggered by cell–cell interactions, including those that may occur between tumor cells and supporting stromal cells. The decreased expression of CTNNA2 in tumors from our poor responders supports a pro-metastatic role for miR-223 as CTNNA2 acts as a tumor suppressor in both endometrial and laryngeal carcinomas (67, 68). Additional evidence for an association between miR-223 and metastasis or chemotherapy resistance has been demonstrated in recurrent ovarian tumors, renal cell metastases, and gastric cancer (69–71).

In contrast, recent reports support a potential tumor suppressor role for miR-223 *in vitro* (72–74). Low miR-223 expression combined with elevated expression of its target gene, epithelial cell transforming sequence 2, in OS tissues is associated with poor outcome (72, 73). It is worth noting that only one of these studies utilized patient tissues, and this included a mix of pediatric and adult tumors (age range 8–66 years) (73). Canine osteosarcoma most closely resembles the pediatric disease, while human adult OS is frequently associated with Paget's disease and may thus involve different underlying molecular mechanisms of progression (1, 75).

Another goal of this study was to integrate miRNA and gene expression data to identify key aberrant pathways contributing to pathogenesis and progression of OS. miRNA–gene expression analysis revealed seven miRNAs with low expression in aggressive tumors predicted or known to target IGF2BP1, an oncogene of interest to our lab. IGF2BP1 has a 3′ UTR that is thousands of kilobases long and with multiple well-conserved binding sites for various miRNAs. This extended 3′ UTR contains multiple polyadenylation sites, with alternate use of polyadenylation sites to produce a shortened 3′ UTR serving as a mechanism by which the gene may avoid miRNA regulation, including at least four sites for the miRNA let-7 (3). We found a statistically significant correlation between low expression of let-7b and increased expression of IGF2BP1 via RT-qPCR in eight OS tumors (Figure 2). Identification of potential miRNA regulators of this protein will facilitate additional functional studies. In addition to IGF2BP1, let-7b targets a variety of oncogenes and has been proposed to act as a tumor suppressor in human osteosarcoma by targeting insulin-like growth factor-1 receptor (76). Despite challenges with effective delivery to target tissues, restoration of tumor-suppressor miRNAs remains a rapidly growing area of research. Studies such as ours may identify new therapeutic miRNAs.

We did not identify any potential interactions between our most dysregulated miRNAs and HES1 nor was Notch signaling identified in our pathway analyses involving miRNAs aberrantly expressed between our DFI cohort tumors. This is consistent with our findings and those of Poos et al. (26) that Notch activation likely contributes to the proliferative response but does not appear to drive metastasis. To further explore the role of miRNAs in Notch activation in OS, we utilized miRNA expression changes identified by comparing tumors to normal bone. We found experimental evidence for interactions between nearly half of the dysregulated miRNAs and one or more Notch/HES1 associated. A handful of these pathways are targetable *via* small molecule inhibitors including Notch, Hedgehog, HER2/ERRB and PARP. Several of these have been or are under investigation for potential use in the treatment of OS (77, 78). Expression studies like ours might identify biomarkers to help stratify patients for optimal therapeutic benefit or monitor therapeutic response.

The last aim of this study was to identify expression changes of presumed tumor-associated miRNAs in the serum associated with patient outcome. Reliable, repeatable RT-qPCR results for measurement of serum are challenging due to typically low miRNA yield, which inhibits efforts to identify and control for poor-quality samples. This has contributed to inconsistencies between circulating biomarker studies and remains a considerable roadblock to the clinical utility and reliability of such screens (79).

Despite these challenges, we set out to identify a data analysis pipeline utilizing the same relatively affordable SYBR green RT-qPCR platform to measure the relative expression of serum miRNAs. We were able to ultimately identify a two-miRNA signature which successfully stratified patients into distinct outcome groups. The most significantly altered miRNA in this signature was miR-30c, which shows a progressively decreased expression from normal bone to tumors and from tumors from dogs with good outcome to tumors from dogs with poor outcome (Supplementary Tables 3–5). While promising, we acknowledge that, for all of our miRNA signatures, predictive capability in an independent tumor set remains to be established.

## Conclusions

In conclusion, we successfully identified miRNA expression changes associated with patient outcome in both OS tumor and patient serum samples. miRNA–gene interactions of the disrupted miRNAs in tumors with genes identified as aberrantly expressed by previous studies (2) can be used to identify targetable pathways disrupted in OS. These studies support the value of miRNA expression studies in biomarker/target discovery efforts for OS.

## Data Availability Statement

The datasets presented in this study can be found in online repositories. The names of the repository/repositories and accession number(s) can be found below: https://www.ncbi.nlm.nih.gov/geo/ (GSE24251).

## Ethics Statement

The animal study was reviewed and approved by Colorado State University Animal Care and Use Committee. Written informed consent was obtained from the owners for the participation of their animals in this study.

## Author Contributions

DDa isolated RNA, conducted RT-qPCR and data analysis, prepared the figures, and wrote the manuscript. AH provided assistance in the statistical analysis of data. GB directed the RT-qPCR analysis of miRNAs. DDu directed the study design and sample acquisition and helped with data analysis and writing and editing of the manuscript. All authors contributed to the article and approved the submitted version.

## Conflict of Interest

The authors declare that the research was conducted in the absence of any commercial or financial relationships that could be construed as a potential conflict of interest.
