# Supplementary material for: MicroRNA Expression Changes and Integrated Pathways Associated With Poor Outcome in Canine Osteosarcoma
Source: Front Vet Sci. 2021 Apr 15;8:637622. doi: 10.3389/fvets.2021.637622 (PMC8081964; doi:10.3389/fvets.2021.637622)
Supplement: Supplementary file 1 [file Data_Sheet_1.docx]

**Table S1**. Patient information for 14 tumors; 7 from dogs with DFI <100 days and 7 from dogs with DFI > 300 days.

| **Unique ID** | **DFI (days)** | **Age at Dx (yrs)** | **Sex** | **Breed** | **Tumor Loc** | **Chemotherapy (doses administered)** |
| --- | --- | --- | --- | --- | --- | --- |
| T1 | 97 | 8.8 | MC | Mix | Femur | Doxorubicin (4) |
| T2 | 80 | 9.4 | MI | Rhodesian | Humerus | Carboplatin (5) |
| T3 | 885 | 8 | FS | Golden | Radius | Carboplatin (3) |
| T4 | 356 | 10.4 | MC | Mix | Radius | Doxorubicin (5) |
| T5 | 40 | 4.4 | MC | Greyhound | Humerus | Doxorubicin (2) |
| T6 | 384 | 11.5 | FS | Mix | Femur | Cisplatin |
| T7 | 90 | 9 | FS | Mix | Tibia | Cisplatin |
| T8 | 467 | 7.1 | MC | Greyhound | Humerus | Doxorubicin (5) |
| T9 | 95 | 10.2 | FS | Labrador | Humerus | Cisplatin |
| T10 | 605 | 7.1 | FS | Greyhound | Femur | Doxorubicin (5) |
| T11 | 77 | 7 | MC | Greyhound | Femur | Carboplatin (3) |
| T12 | 466 | 6.9 | MC | Rottweiler | Radius | Doxo(2) Carbo (2) |
| T13 | 94 | 8 | FS | Greyhound | Tibia | Doxorubicin (4) |
| T14 | 307 | 7.6 | FS | Mix | Radius | Cisplatin |

**Table S2.** Patient data for 33 canine OS patients.

| **Unique ID** | **DFI (days)** | **Age at Dx (yrs)** | **Sex** | **Breed** | **Tumor Location** | **Chemotherapy (doses administered)** |
| --- | --- | --- | --- | --- | --- | --- |
| 6B | 97 | 8.8 | MC | Mix | Femur | Doxorubicin (4) |
| 11B | 252 | 7.5 | FS | Mastif | Radius | Doxorubicin (1) |
| 25B | 466 | 6.9 | MC | Rottweiler | Radius | Doxo (2) Carbo (2) |
| 60B | 427 | 6.5 | MI | Old Eng Sheep | Tibia | Doxo (5) Carbo (5) |
| 41B | 80 | 9.4 | MI | Rhodesian | Humerus | Carboplatin (3) |
| 5B | 120 | 6.6 | MC | Mix | Radius | Doxo (3) Carbo (3) |
| 4B | 406 | 9.4 | MC | Mix | Radius | Doxorubicin (5) |
| 7B | 150 | 10.8 | FS | Golden Ret | Humerus | Doxorubicin (5) |
| 9B | 232 | 6.6 | FS | Dane | Humerus | Doxo (2) Carbo (3) |
| 51B | 34 | 6.1 | F | Dane | Tibia | Carboplatin (2) |
| 17B | 475 | 9.5 | MC | Husky | Femur | Doxo (3) Carbo (3) |
| 52B | 95 | 11.8 | FS | Mix | Humerus | Doxorubicin (4) |
| 18B | 151 | 6.9 | MC | Mix | Femur | Doxo (3) Carbo (3) |
| 20B | 937 | 10.4 | MC | Australian Shep | Tibia | Doxo (3) Carbo (3) |
| 22B | 605 | 7.1 | FS | Greyhound | Femur | Doxorubicin (5) |
| 23B | 20 | 9.8 | MC | Germ Short Point | Radius | Carboplatin (1) |
| 24B | 218 | 5.1 | MC | Pyrenees | Radius | Doxo (3) Carbo (3) |
| 54B | 127 | 4.8 | MC | Pyrenees | Radius | Doxo (2), Carbo (3), & Cisp (2) |
| 55B | 91 | 6.1 | FS | Greyhound | Humerus | Doxo (2) Carbo (3) |
| 30B | 296 | 11.4 | MC | Labrador Ret | Scapula | Carboplatin (4) |
| 32B | 299 | 8.0 | FS | Dane | Radius | Carboplatin (2) |
| 33B | 246 | 7.9 | MC | Rottweiler | Tibia | Doxorubicin (4) |
| 34B | 64 | 7.5 | FS | Rottweiler | Humerus | Doxo (1) Carbo (1) |
| 35B | 190 | 8.5 | MC | Labrador Ret | Radius | Doxo (3) Carbo (3) |
| 36B | 75 | 5.3 | MI | Leonburger | Tibia, tallus | Doxo (1) Carbo (2) |
| 59B | 392 | 11.1 | FS | Mix | Tibia | Carboplatin (3) |
| 37B | 132 | 4.2 | FS | Saint Bernard | Radius | Carboplatin (4) |
| 38B | 256 | 10.8 | MC | Great Pyrenees | Radius | Doxo (2) Carbo (2) |
| 40B | 216 | 8.5 | MC | Rottweiler | Femur | Carboplatin (4) |
| 61B | 97 | 8.7 | FS | Flat-Coated Ret | Radius | Doxo (4) Carbo (4) |
| 42B | 77 | 7.2 | MC | Labrador Ret | Radius | Doxo (2) Carbo (1) |
| 43B | 756 | 8.9 | FS | Coonhound | Radius | Carboplatin (5) |
| 44B | 376 | 7.1 | MC | Labrador Ret | Humerus | Carboplatin (6) |

**Table S3**: Differentially expressed miRNAs in tumors from dogs with poor response (DFI<100 days) compared to tumors from dogs with good response (DFI>300 days).

| **miRName** | **Ttest p-val** | **Fold Change** | **Direction of Exp Δ** |
| --- | --- | --- | --- |
| **miR-26a-5p** | **0.0203** | **-2.00** | **down** |
| **miR-142-3p** | **0.0281** | **2.77** | **up** |
| **miR-135a-3p** | **0.0324** | **-9.76** | **down** |
| **miR-451a** | **0.0492** | **4.45** | **up** |
| **miR-30c-5p** | **0.0564** | **-1.61** | **down** |
| let-7c-5p | 0.0593 | -1.76 | down |
| **let-7b-5p** | **0.0604** | **-2.07** | **down** |
| **miR-181b-5p** | **0.0670** | **-2.20** | **down** |
| **miR-128-3p** | **0.0710** | **-2.00** | **down** |
| **miR-16-5p** | **0.0735** | **1.47** | **up** |
| **miR-196b-5p** | **0.0780** | **-2.04** | **down** |
| **miR-17-5p** | **0.0780** | **1.61** | **up** |
| **miR-223-3p** | **0.0829** | **4.02** | **up** |
| **miR-7-5p** | **0.0901** | **-2.66** | **down** |
| miR-520d-3p | 0.0960 | -2.27 | down |
| miR-196a-5p | 0.0961 | -1.91 | down |
| miR-376b-3p | 0.1051 | -3.33 | down |
| miR-181d-5p | 0.1071 | -1.95 | down |
| **miR-130a-5p** | **0.1086** | **-1.25** | **down** |
| **miR-206** | **0.1106** | **3.25** | **up** |
| **miR-18a-5p** | **0.1109** | **2.00** | **up** |
| miR-18b-5p | 0.1123 | 1.97 | up |
| let-7a-5p | 0.1149 | -1.31 | down |
| **miR-210-3p** | **0.1168** | **1.64** | **up** |
| **miR-9-5p** | **0.1190** | **-2.45** | **down** |
| miR-135b-5p | 0.1291 | -2.95 | down |
| miR-181a-2-3p | 0.1325 | -1.57 | down |
| **miR-199a-5p** | **0.1363** | **-1.42** | **down** |
| miR-421 | 0.1388 | 1.54 | up |
| miR-34c-5p | 0.1448 | -1.91 | down |
| miR-155-5p | 0.1535 | 1.92 | up |
| miR-142-5p | 0.1554 | 2.70 | up |
| let-7g-5p | 0.1557 | 1.28 | up |
| miR-106a-5p | 0.1596 | 1.52 | up |
| miR-18b-3p | 0.1633 | -1.68 | down |
| miR-519e-3p | 0.1698 | -1.75 | down |
| miR-19b-3p | 0.1712 | 1.57 | up |
| miR-199b-5p | 0.1729 | -1.38 | down |
| miR-125a-5p | 0.1808 | -1.48 | down |
| miR-200a-3p | 0.1817 | 2.00 | up |
| miR-214-3p | 0.1878 | -1.64 | down |
| miR-200b-3p | 0.1953 | -1.76 | down |
| miR-181a-5p | 0.1967 | -1.87 | down |
| miR-519a-3p | 0.1999 | -2.56 | down |

**Table S4**: MicroRNAs up-regulated in tumors relative to normal bone.

| **miR Name** | **Ttest P-val** | **Fold Change** |
| --- | --- | --- |
| miR-7-5p | 2.5429E-08 | 24.58 |
| miR-9-5p | 0.0038 | 23.34 |
| miR-346 | 0.0154 | 12.52 |
| miR-96-5p | 0.0001 | 8.11 |
| miR-382-5p | 0.0302 | 7.36 |
| miR-663a | 0.0000 | 7.08 |
| miR-654-3p | 0.0003 | 6.46 |
| miR-493-3p | 0.0132 | 5.75 |
| miR-92b-3p | 0.0027 | 5.19 |
| miR-205-5p | 0.0003 | 5.13 |
| miR-18b-3p | 0.0000 | 4.23 |
| miR-135b-5p | 0.0013 | 4.14 |
| miR-132-3p | 0.0005 | 4.01 |
| miR-214-3p | 0.0043 | 3.51 |
| miR-199b-5p | 0.0036 | 2.94 |
| miR-331-5p | 0.0347 | 2.50 |
| miR-199a-5p | 0.0256 | 2.29 |
| miR-21-5p | 0.0074 | 2.17 |
| miR-328-3p | 0.0012 | 2.08 |

**Table S5**: MicroRNAs with lower expression in tumors compared to normal bone.

| **miR Name** | **Ttest P-val** | **Fold Change** |
| --- | --- | --- |
| miR-107 | 3.7831E-08 | -74.08 |
| miR-133b | 0.0068 | -24.70 |
| miR-141-3p | 0.0012 | -23.92 |
| miR-206 | 0.0124 | -20.20 |
| miR-223-3p | 0.0091 | -18.72 |
| miR-208-3p | 0.0023 | -17.97 |
| miR-133a-3p | 0.0231 | -11.65 |
| miR-150-5p | 0.0002 | -6.86 |
| miR-146b-5p | 0.0020 | -5.11 |
| miR-26a-5p | 0.0025 | -4.47 |
| miR-29c-3p | 0.0015 | -3.64 |
| miR-26b-5p | 0.0000 | -3.60 |
| miR-129-5p | 0.0310 | -3.12 |
| miR-34a-5p | 0.0002 | -2.94 |
| miR-125b-5p | 0.0065 | -2.70 |
| miR-29a-3p | 0.0036 | -2.60 |
| miR-146a-5p | 0.0010 | -2.55 |
| miR-30c-5p | 0.0005 | -2.52 |
| miR-106b-5p | 0.0066 | -2.49 |
| miR-99a-5p | 0.0085 | -2.29 |
| miR-100-5p | 0.0304 | -2.09 |

**Table S7**: miRNA-mRNA interactions between dysregulated miRNAs and mRNAs in tumors from dogs with short DFI as determined by multiMiR *in silico* analysis.

| **Upregulated miRNAs and Downregulated Genes** | | |
| --- | --- | --- |
| **miRNA.ID** | **Target.Gene** | **Evidence** |
| hsa-miR-142-3p | KIAA1191 | Validated |
| hsa-miR-17-5p | SEPT11 | Validated |
| hsa-miR-142-3p | ARID5B | Predicted |
| hsa-miR-142-3p | JAZF1 | Predicted |
| hsa-miR-142-3p | EBF1 | Predicted |
| hsa-miR-17-5p | CXCL14 | Predicted |
| hsa-miR-17-5p | JAZF1 | Predicted |
| hsa-miR-17-5p | CAMK2N1 | Predicted |
| hsa-miR-17-5p | CCDC73 | Predicted |
| hsa-miR-17-5p | KIAA1191 | Predicted |
| hsa-miR-17-5p | IPO9 | Predicted |
| **hsa-miR-223-3p** | **DST** | **Predicted** |
| **hsa-miR-223-3p** | **CTNNA2** | **Predicted** |
| **Downregulated miRNAs and Upregulated Genes** | | |
| **miRNA.ID** | **Target.Gene** | **Evidence** |
| **hsa-let-7b-5p** | **IGF2BP1** | **Validated** |
| hsa-miR-26a-5p | OLA1 | Validated |
| hsa-let-7b-5p | PDZRN4 | Predicted |
| hsa-let-7b-5p | DSCAM | Predicted |
| hsa-miR-130a-3p | GDA | Predicted |
| **hsa-miR-130a-3p** | **IGF2BP1** | **Predicted** |
| hsa-miR-135a-5p | GRIA4 | Predicted |
| **hsa-miR-135a-5p** | **IGF2BP1** | **Predicted** |
| hsa-miR-181b-5p | PDE10A | Predicted |
| hsa-miR-181b-5p | GDA | Predicted |
| hsa-miR-181b-5p | RAB11FIP1 | Predicted |
| **hsa-miR-196b-5p** | **IGF2BP1** | **Predicted** |
| hsa-miR-196b-5p | RANBP3L | Predicted |
| hsa-miR-196b-5p | DSCAM | Predicted |
| hsa-miR-199a-5p | GRIA4 | Predicted |
| **hsa-miR-199a-5p** | **IGF2BP1** | **Predicted** |
| hsa-miR-26a-5p | GRIA4 | Predicted |
| **hsa-miR-26a-5p** | **IGF2BP1** | **Predicted** |
| **hsa-miR-9-5p** | **IGF2BP1** | **Predicted** |

**Bold interactions are discussed in the text.**

**Table S8.** Primers used for miRNA RT-qPCR.

| **miRbase Name (v.21)** | **Primer Sequence** |
| --- | --- |
| hsa-let-7a-5p | TGAGGTAGTAGGTTGTATAGTT |
| hsa-let-7b-5p | TGAGGTAGTAGGTTGTGTGGTT |
| hsa-let-7c-5p | TGAGGTAGTAGGTTGTATGGTT |
| hsa-let-7d-5p | AGAGGTAGTAGGTTGCATAGTT |
| hsa-let-7e-5p | TGAGGTAGGAGGTTGTATAGTT |
| hsa-let-7f-5p | TGAGGTAGTAGATTGTATAGTT |
| hsa-let-7g-5p | TGAGGTAGTAGTTTGTACAGTT |
| hsa-let-7i-5p | TGAGGTAGTAGTTTGTGCGTT |
| hsa-miR-1-3p | TGGAATGTAAAGAAGTATGTAT |
| hsa-miR-100-5p | AACCCGTAGATCCGAACTTGTG |
| hsa-miR-101-3p | TACAGTACTGTGATAACTGAA |
| hsa-miR-106a-5p | AAAAGTGCTTACAGTGCAGGTAG |
| hsa-miR-106b-5p | TAAAGTGCTGACAGTGCAGAT |
| hsa-miR-107 | AGCAGCATTGTACAGGGCTATCA |
| hsa-miR-122-5p | TGGAGTGTGTGACAATGGTGTTTG |
| hsa-miR-125a-5p | TCCCTGAGACCCTTTAACCTGTGA |
| hsa-miR-125b-5p | TCCCTGAGACCCTAACTTGTGA |
| hsa-miR-128-3p | TCACAGTGAACCGGTCTCTTT |
| hsa-miR-128-2-5p | GGGGGCCGATACACTGTACGAGA |
| hsa-miR-129-5p | CTTTTTGCGGTCTGGGCTTGC |
| hsa-miR-130a-3p | CAGTGCAATGTTAAAAGGGCAT |
| hsa-miR-130b-3p | CAGTGCAATGATGAAAGGGCAT |
| hsa-miR-132-3p | CAGTGCAATGATGAAAGGGCAT |
| hsa-miR-133a-3p | TTTGGTCCCCTTCAACCAGCTG |
| hsa-miR-133b | TTTGGTCCCCTTCAACCAGCTA |
| hsa-miR-135a-5p | TATGGCTTTTTATTCCTATGTGA |
| hsa-miR-135b-5p | TATGGCTTTTCATTCCTATGTGA |
| hsa-miR-138-5p | AGCTGGTGTTGTGAATCAGGCCG |
| hsa-miR-141-3p | TAACACTGTCTGGTAAAGATGG |
| hsa-miR-142-3p | TGTAGTGTTTCCTACTTTATGGA |
| hsa-miR-142-5p | CATAAAGTAGAAAGCACTACT |
| hsa-miR-145-5p | GTCCAGTTTTCCCAGGAATCCCT |
| hsa-miR-146a-5p | TGAGAACTGAATTCCATGGGTT |
| hsa-miR-146b-5p | TGAGAACTGAATTCCATAGGCT |
| hsa-miR-148a-3p | TCAGTGCACTACAGAACTTTGT |
| hsa-miR-148b-3p | TCAGTGCATCACAGAACTTTGT |
| hsa-miR150-5p | TCTCCCAACCCTTGTACCAGTG |
| hsa-miR-152-3p | TCAGTGCATGACAGAACTTGG |
| hsa-miR-155-5p | TTAATGCTAATCGTGATAGGGGT |
| hsa-miR-15a-5p | TAGCAGCACATAATGGTTTGTG |
| hsa-miR-15b-5p | TAGCAGCACATCATGGTTTACA |
| hsa-miR-16-5p | TAGCAGCACGTAAATATTGGCG |
| hsa-miR-17-5p | CAAAGTGCTTACAGTGCAGGTAG |
| hsa-miR-181a-2-3p | ACCACTGACCGTTGACTGTACC |
| hsa-miR-181a-5p | AACATTCAACGCTGTCGGTGAGT |
| hsa-miR-181b-5p | AACATTCATTGCTGTCGGTGGGT |
| hsa-miR-181c-5p | AACATTCAACCTGTCGGTGAGT |
| hsa-miR-181d-5p | AACATTCATTGTTGTCGGTGGGT |
| hsa-miR-182-5p | TTTGGCAATGGTAGAACTCACACT |
| hsa-miR-185-5p | TGGAGAGAAAGGCAGTTCCTGA |
| hsa-miR 186-5p | CAAAGAATTCTCCTTTTGGGCT |
| hsa-miR-18a-5p | TAAGGTGCATCTAGTGCAGATAG |
| hsa-miR-18b-5p | TAAGGTGCATCTAGTGCAGTTAG |
| hsa-miR-18b-3p | TGCCCTAAATGCCCCTTCTGGC |
| hsa-miR-192-5p | CTGACCTATGAATTGACAGCC |
| hsa-miR-194-5p | TGTAACAGCAACTCCATGTGGA |
| hsa-miR-195-5p | TAGCAGCACAGAAATATTGGC |
| hsa-miR-196a-5p | TAGGTAGTTTCATGTTGTTGGG |
| hsa-miR-196b-5p | TAGGTAGTTTCCTGTTGTTGGG |
| hsa-miR-199a-5p | CCCAGTGTTCAGACTACCTGTTC |
| hsa-miR-199b-5p | CCCAGTGTTTAGACTATCTGTTC |
| hsa-miR-200a-3p | TAACACTGTCTGGTAACGATGT |
| hsa-miR-200b-3p | TAATACTGCCTGGTAATGATGA |
| hsa-miR-200c-3p | TAATACTGCCGGGTAATGATGGA |
| hsa-miR-202-3p | AGAGGTATAGGGCATGGGAA |
| hsa-miR-203a-3p | GTGAAATGTTTAGGACCACTAG |
| hsa-miR-205-5p | TCCTTCATTCCACCGGAGTCTG |
| hsa-miR-206 | TGGAATGTAAGGAAGTGTGTGG |
| hsa-miR-208a-3p | ATAAGACGAGCAAAAAGCTTGT |
| hsa-miR-208b-3p | ATAAGACGAACAAAAGGTTTGT |
| hsa-miR-20a-5p | TAAAGTGCTTATAGTGCAGGTAG |
| hsa-miR-20b-5p | CAAAGTGCTCATAGTGCAGGTAG |
| hsa-miR-21-5p | TAGCTTATCAGACTGATGTTGA |
| hsa-miR-210-3p | CTGTGCGTGTGACAGCGGCTGA |
| hsa-miR-212-3p | TAACAGTCTCCAGTCACGGCC |
| hsa-miR-214-3p | ACAGCAGGCACAGACAGGCAGT |
| hsa-miR-217 | TACTGCATCAGGAACTGATTGGA |
| hsa-miR-218-5p | TTGTGCTTGATCTAACCATGT |
| hsa-miR-22-3p | AAGCTGCCAGTTGAAGAACTGT |
| hsa-miR-221-3p | AGCTACATTGTCTGCTGGGTTTC |
| hsa-miR-222-3p | AGCTACATCTGGCTACTGGGT |
| hsa-miR-223-3p | TGTCAGTTTGTCAAATACCCCA |
| hsa-miR-224-5p | CAAGTCACTAGTGGTTCCGTT |
| hsa-miR-224-3p | AAAATGGTGCCCTAGTGACTACA |
| hsa-miR-23a-3p | ATCACATTGCCAGGGATTTCC |
| hsa-miR-23b-3p | ATCACATTGCCAGGGATTACC |
| hsa-miR-24-3p | TGGCTCAGTTCAGCAGGAACAG |
| hsa-miR-25-3p | CATTGCACTTGTCTCGGTCTGA |
| hsa-miR-26a-5p | TTCAAGTAATCCAGGATAGGCT |
| hsa-miR-26b-5p | TTCAAGTAATTCAGGATAGGT |
| hsa-miR-27a-3p | TTCACAGTGGCTAAGTTCCGC |
| hsa-miR-27b-3p | TTCACAGTGGCTAAGTTCTGC |
| hsa-miR-28-5p | AAGGAGCTCACAGTCTATTGAG |
| hsa-miR-296-5p | AGGGCCCCCCCTCAATCCTGT |
| hsa-miR-29a-3p | TAGCACCATCTGAAATCGGTTA |
| hsa-miR-29b-3p | TAGCACCATTTGAAATCAGTGTT |
| hsa-miR-29c-3p | TAGCACCATTTGAAATCGGTTA |
| hsa-miR-30a-5p | TGTAAACATCCTCGACTGGAAG |
| hsa-miR-30b-5p | TGTAAACATCCTACACTCAGCT |
| hsa-miR-30c-5p | TGTAAACATCCTACACTCTCAGC |
| hsa-miR-30d-5p | TGTAAACATCCCCGACTGGAAG |
| hsa-miR-30e-5p | TGTAAACATCCTTGACTGGAAG |
| hsa-miR-31-5p | AGGCAAGATGCTGGCATAGCT |
| hsa-miR-32-5p | TATTGCACATTACTAAGTTGCA |
| hsa-miR-320a | AAAAGCTGGGTTGAGAGGGCGA |
| hsa-miR-320b | AAAAGCTGGGTTGAGAGGGCAA |
| hsa-miR-320c | AAAAGCTGGGTTGAGAGGGT |
| hsa-miR-320d | AAAAGCTGGGTTGAGAGGA |
| hsa-miR-326 | CCTCTGGGCCCTTCCTCCAG |
| hsa-miR-328-3p | CTGGCCCTCTCTGCCCTTCCGT |
| hsa-miR-331-3p | GCCCCTGGGCCTATCCTAGAA |
| hsa-miR-331-5p | CTAGGTATGGTCCCAGGGATCC |
| hsa-miR-335-5p | TCAAGAGCAATAACGAAAAATGT |
| hsa-miR-337-3p | CTCCTATATGATGCCTTTCTTC |
| hsa-miR-337-5p | GAACGGCTTCATACAGGAGTT |
| hsa-miR-339-5p | TCCCTGTCCTCCAGGAGCTCACG |
| hsa-miR-340-5p | TTATAAAGCAATGAGACTGATT |
| hsa-miR-346 | TGTCTGCCCGCATGCCTGCCTCT |
| hsa-miR-34a-5p | TGGCAGTGTCTTAGCTGGTTGT |
| hsa-miR-34b-3p | CAATCACTAACTCCACTGCCAT |
| hsa-miR-34b-5p | TAGGCAGTGTCATTAGCTGATTG |
| hsa-miR-34c-5p | AGGCAGTGTAGTTAGCTGATTGC |
| hsa-miR-370-3p | GCCTGCTGGGGTGGAACCTGGT |
| hsa-miR-371a-5p | ACTCAAACTGTGGGGGCACT |
| hsa-miR-373-3p | GAAGTGCTTCGATTTTGGGGTGT |
| hsa-miR-374a-5p | TTATAATACAACCTGATAAGTG |
| hsa-miR-374b-5p | ATATAATACAACCTGCTAAGTG |
| hsa-miR-376a-3p | ATCATAGAGGAAAATCCACGT |
| hsa-miR-376b-3p | ATCATAGAGGAAAATCCATGTT |
| hsa-miR-376c-3p | AACATAGAGGAAATTCCACGT |
| hsa-miR-377-3p | ATCACACAAAGGCAACTTTTGT |
| hsa-miR-318-3p | TATACAAGGGCAAGCTCTCTGT |
| hsa-miR-421 | ATCAACAGACATTAATTGGGCGC |
| hsa-miR-422a | ACTGGACTTAGGGTCAGAAGGC |
| hsa-miR-424-3p | CAAAACGTGAGGCGCTGCTAT |
| hsa-miR-424-5p | CAGCAGCAATTCATGTTTTGAA |
| hsa-miR-429 | TAATACTGTCTGGTAAAACCGT |
| hsa-miR-431-5p | TGTCTTGCAGGCCGTCATGCA |
| hsa-miR-451a | AAACCGTTACCATTACTGAGTT |
| hsa-miR-454-3p | TAGTGCAATATTGCTTATAGGGT |
| hsa-miR-484 | TCAGGCTCAGTCCCCTCCCGAT |
| hsa-miR-493-3p | TGAAGGTCTACTGTGTGCCAGG |
| hsa-miR-495-3p | AAACAAACATGGTGCACTTCTT |
| hsa-miR-497-5p | CAGCAGCACACTGTGGTTTGT |
| hsa-miR-503-5p | TAGCAGCGGGAACAGTTCTGCAG |
| hsa-miR-513a-3p | TAAATTTCACCTTTCTGAGAAGG |
| hsa-miR-513b-5p | TTCACAAGGAGGTGTCATTTAT |
| hsa-miR-513c-5p | TTCTCAAGGAGGTGTCGTTTAT |
| hsa-miR-519a-3p | AAAGTGCATCCTTTTAGAGTGT |
| hsa-miR-519b-3p | AAAGTGCATCCTTTTAGAGGTT |
| hsa-miR-519c-3p | AAAGTGCATCTTTTTAGAGGAT |
| hsa-miR-519-3p | CAAAGTGCCTCCCTTTAGAGTG |
| hsa-miR-519e-3p | AAGTGCCTCCTTTTAGAGTGTT |
| hsa-miR-520a-3p | AAAGTGCTTCCCTTTGGACTGT |
| hsa-miR-520b | AAAGTGCTTCCTTTTAGAGGG |
| hsa-miR-520c-3p | CTCTAGAGGGAAGCACTTTCTG |
| hsa-miR-520d-3p | AAAGTGCTTCTCTTTGGTGGGT |
| hsa-miR-520e | AAAGTGCTTCCTTTTTGAGGG |
| hsa-miR-520f-3p | AAGTGCTTCCTTTTAGAGGGTT |
| hsa-miR-520g-3p | ACAAAGTGCTTCCCTTTAGAGTGT |
| hsa-miR-520h | ACAAAGTGCTTCCCTTTAGAGT |
| hsa-miR-551a | GCGACCCACTCTTGGTTTCCA |
| hsa-miR-551b-3p | GCGACCCATACTTGGTTTCAG |
| hsa-miR-590-3p | TAATTTTATGTATAAGCTAGT |
| hsa-miR-630 | AGTATTCTGTACCAGGGAAGGT |
| hsa-miR-654-3p | TATGTCTGCTGACCATCACCTT |
| hsa-miR-657 | GGCAGGTTCTCACCCTCTCTAGG |
| hsa-miR-663a | AGGCGGGGCGCCGCGGGACCGC |
| hsa-miR-7-5p | TGGAAGACTAGTGATTTTGTTGT |
| hsa-miR-9-5p | TCTTTGGTTATCTAGCTGTATGA |
| hsa-miR-96-5p | TTTGGCACTAGCACATTTTTGCT |
| hsa-miR-96-3p | AATCATGTGCAGTGCCAATATG |
| hsa-miR-98-5p | TGAGGTAGTAAGTTGTATTGTT |
| hsa-miR-99b-5p | CACCCGTAGAACCGACCTTGCG |
| hsa-miR-99a-5p | AACCCGTAGATCCGATCTTGTG |
| hsa-miR-10a-5p | TACCCTGTAGATCCGAATTTGTG |
| hsa-miR-10b-5p | TACCCTGTAGAACCGAATTTGTG |
| hsa-miR-19a-3p | TGTGCAAATCTATGCAAAACTGA |
| hsa-miR-19b-3p | TGTGCAAATCCATGCAAAACTGA |
| hsa-miR-92a-3p | TATTGCACTTGTCCCGGCCTGT |
| hsa-miR-92b-3p | TATTGCACTCGTCCCGGCCTCC |
| hsa-miR-134-5p | TGTGACTGGTTGACCAGAGGGG |
| hsa-miR-154-5p | TAGGTTATCCGTGTTGCCTTCG |
| hsa-miR-301a-3p | CAGTGCAATAGTATTGTCAAAGC |
| hsa-miR-301b-3p | CAGTGCAATGATATTGTCAAAGC |
| hsa-miR-369-3p | AATAATACATGGTTGATCTTT |
| hsa-miR-382-5p | GAAGTTGTTCGTGGTGGATTCG |
| hsa-miR-544a | ATTCTGCATTTTTAGCAAGTTC |
